# Crystal structure of (*E*)-*N*-{2-[2-(4-methyl­benzyl­idene)hydrazin-1-yl]-2-oxoeth­yl}-*p*-toluene­sulfonamide

**DOI:** 10.1107/S2056989015009330

**Published:** 2015-05-30

**Authors:** H. Purandara, Sabine Foro, B. Thimme Gowda

**Affiliations:** a Department of Chemistry, Mangalore University, Mangalagangotri 574 199, Mangalore, India; b Institute of Materials Science, Darmstadt University of Technology, Alarich-Weiss-Strasse 2, D-64287 Darmstadt, Germany; c Bangalore University, Jnanabharati, Bangalore 560 056, India

**Keywords:** crystal structure, *p*-toluene­lsulfon­yl, glycin­yl, aryl­hydrazone, hydrogen bonding

## Abstract

The title acyl­hydrazone derivative, C_17_H_19_N_3_O_3_S, containing an amino acid moiety and electron-donating substituents attached to both the phenyl rings, crystallized with two independent mol­ecules (*A* and *B*) in the asymmetric unit. The mol­ecules are bent at the S atom, with C—SO_2_—NH—CH_2_ torsion angles of −67.3 (2) and 67.7 (3)° in mol­ecules *A* and *B*, respectively. Further, the dihedral angles between the sulfonyl­glycine segments and the *p*-toluene­sulfonyl rings are 76.1 (1) and 85.8 (1)° in mol­ecules *A* and *B*, respectively. The central hydrazone segments and the toluene rings attached to them are almost co-planar with their mean planes being inclined to one another by 5.2 (2) (mol­ecule *A*) and 2.9 (2)° (mol­ecule *B*). The dihedral angles between the benzene rings are 86.83 (12) (mol­ecule *A*) and 74.00 (14)° (mol­ecule *B*). In the crystal, the *A* mol­ecules are linked by a pair of N—H⋯O hydrogen bonds, forming inversion dimers with an *R*_2_^2^(8) ring motif. The dimers are linked *via* three N—H⋯O hydrogen bonds involving the *B* mol­ecules, forming chains along [100] and enclosing *R*_2_^2^(12) and *R*_4_^4^(16) ring motifs. The chains are linked *via* C—H⋯O hydrogen bonds and a C—H⋯π inter­action, forming sheets parallel to (010). There is a further C—H⋯π inter­action and a slipped parallel π–π inter­action [inter-centroid distance = 3.8773 (16) Å] between the sheets, leading to the formation of a three-dimensional framework.

## Abstract 2

The title compound, an aryl­sulfonyl glycinyl aryl hydrazone Schiff base, crystallizes with two independent mol­ecules in the asymmetric unit. In the crystal, a series of N—H⋯O and C—H⋯O hydrogen bonds and C—H⋯π and slipped parallel π–π inter­actions link the mol­ecules, forming a three-dimensional structure.

## Chemical context   

Hydrazones display numerous biological activities. The hydrazone Schiff bases of aroyl, acyl and heteroaroyl compounds are more versatile and flexible (in the sense that they can be used as reaction intermediates in organic synthesis and as ligands forming complexes with metal ions in coordination chemistry) due to the presence of the C=O group, an additional donor site. *N*-acyl­hydrazones containing a glycine residue have been investigated extensively for their biological and medical activities (Tian *et al.*, 2011 ▸). Anti­viral activity has been shown for acyl­hydrazone derivatives which contain an amino acid moiety and an electron-donating substituent in the sulfonyl phenyl ring (Tian *et al.*, 2009 ▸). The biological activities of these Schiff bases are thought to be related to structural aspects. 

In a continuation of our studies of substituent effects on the structures of such compounds, for example *N*-(ar­yl)-amides (Gowda *et al.*, 2006 ▸; Rodrigues *et al.*, 2011 ▸), *N*-chloro­aryl­amides (Jyothi & Gowda, 2004 ▸) and *N*-bromo­aryl­sulfonamides (Usha & Gowda, 2006 ▸), we report herein on the synthesis and crystal structure of the title compound. This acyl­hydrazone derivative contains a glycine moiety and electron-donating substituents in both the sulfonyl and hydrazone aromatic rings.
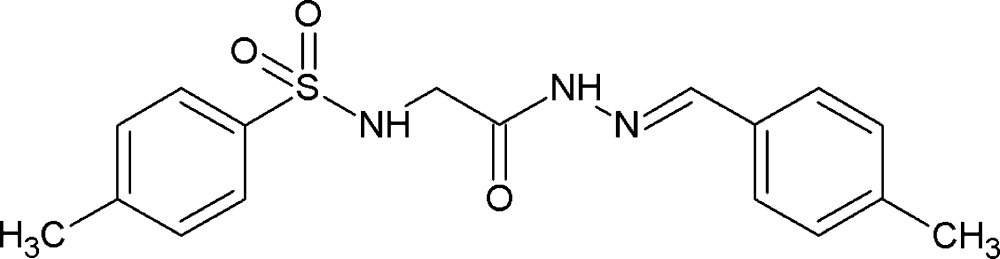


## Structural commentary   

**Figure 1:**
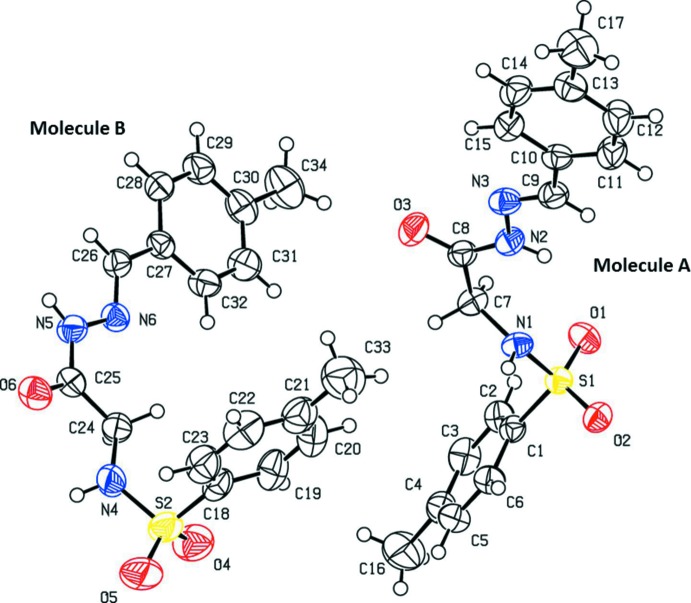
The mol­ecular structure of the two independent mol­ecules of the title compound, showing the atom labelling. Displacement ellipsoids are drawn at the 50% probability level.

The mol­ecular structures of the two independent mol­ecules (*A* and *B*) of the title compound are shown in Fig. 1 ▸. It can be seen quite clearly from Fig. 1 ▸ that mol­ecule *A* has an extended conformation while mol­ecule *B* is U-shaped. In mol­ecule *A*, the conformations of the hydrazide N—H and C—H bonds are *syn* to each other, while the N—H and C=O bonds are *anti* to each other. On the sulfonamide side, the conformations of the sulfonamide N—H and C=O bonds are *syn* to each other. In mol­ecule *B*, the conformations of the hydrazide N—H and C—H bonds, the hydrazide N—H and C=O, and the C=O and sulfonamide N—H bonds are all *syn* to each other.

In mol­ecule *A*, the benzene rings are inclined to one another by 86.83 (12)°. The mean plane through atoms C9/N3/N2/C8/O3/C7 [maximum deviation of 0.043 (2) Å for N2], the central section of the mol­ecule, is inclined to the two benzene rings, C1–C6 and C10–C15, by 86.38 (12) and 7.22 (12)°, respectively. In mol­ecule *B*, the benzene rings (C18–C23 and C27–C32) are inclined to one another by 74.00 (14)°, and by 76.85 (13) and 2.91 (12)°, respectively, to the mean plane through atoms C26/N6/N5/C25/O6/C24 [maximum deviation of 0.061 (2) Å for C26]. The different conformations of mol­ecules *A* and *B* are further demonstrated by the differences in the equivalent torsion angles; N1—C7—C8—N2 = 29.3 (3) ° in *A*, compared to N4—C24—C25—N5 = 177.2 (2)° in *B*, and C1—S1—N1—C7 = −67.3 (2)° in *A*, compared to C18—S2—N4—C24 = 67.7 (3)° in *B*.

The carbonyl bonds lengths, C8—O3 in *A* and C25—O6 in *B*, are 1.214 (3) and 1.229 (3) Å, respectively, indicating that the mol­ecules exist in the keto form in the solid state. The C9=N3 and C26=N6 bond lengths, both 1.272 (3) Å in mol­ecules *A* and *B*, respectively, confirm their significant double-bond character. The N2—N3 and N5—N6 bond distances are 1.383 (3) and 1.379 (3) Å, respectively, and the C8—N2 and C25—N5 bond distances are 1.339 (3) and 1.334 (3) Å, respectively, which indicates significant delocalization of π-electron density over the hydrazone portions of the mol­ecules.

## Supra­molecular features   

**Figure 2:**
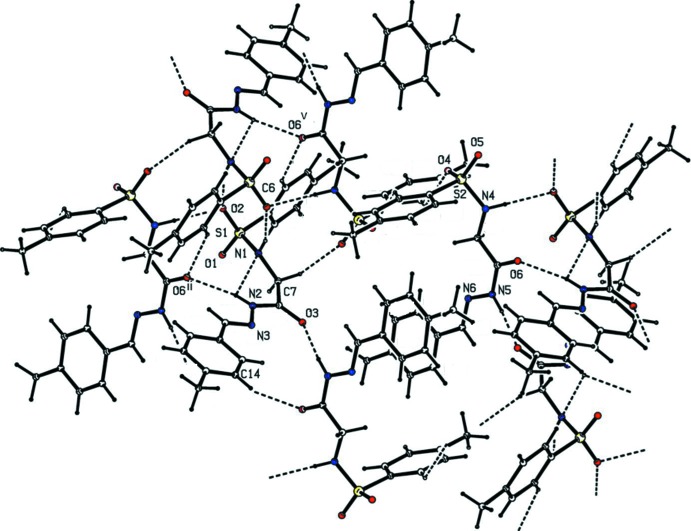
Hydrogen-bonding pattern in the title compound (see Table 1 ▸ for details).

**Figure 3:**
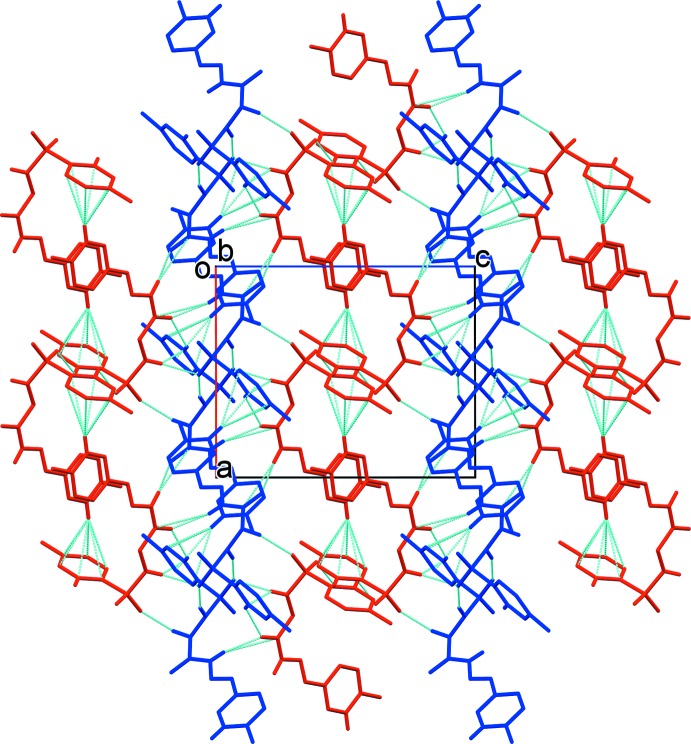
A view along the *b* axis of the crystal packing of the title compound. For details of the hydrogen bonds and C—H⋯π inter­actions (dashed lines), see Table 1 ▸ (mol­ecule *A* is blue and mol­ecule *B* is red).

In the crystal, the *A* mol­ecules are linked by a pair of N—H⋯O hydrogen bonds, forming inversion dimers with an 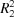
(8) ring motif. The dimers are linked *via* three N—H⋯O hydrogen bonds involving the *B* mol­ecules, forming chains along [100] that enclose 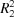
(12) and 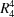
(16) ring motifs (Table 1 ▸ and Fig. 2 ▸). The chains are linked *via* C—H⋯O hydrogen bonds and a C—H⋯π inter­action, forming sheets parallel to (010). The is a C—H⋯π inter­action and a slipped parallel π–π inter­action [*Cg*2⋯*Cg*2^i^ = 3.8773 (16) Å; inter-planar distance = 3.6071 (11) Å; slippage = 1.422 Å; *Cg*2 is the centroid of ring C10–C15, symmetry code: (i) −*x*, −*y* + 1, −*z*], between the sheets, leading to the formation of a three-dimensional framework (Fig. 3 ▸).

## Database survey   

A search of the Cambridge Structural Database (Version 5.36; Groom & Allen, 2014 ▸) for the fragment –NH–CH_2_–C(=O)–NH–N=CH– yielded only one hit, namely *N*-(2-hy­droxy-1-naphthyl­methyl­ene)-*N*′-(*N*-phenyl­glyc­yl)hydrazine (MEMTOO; Gudasi *et al.*, 2006 ▸). We have also very recently reported the crystal structure of a similar compound, namely (*E*)-*N*-{2-[2-(3-chloro­benzyl­idene) hydrazin­yl]-2-oxoeth­yl}-4-methyl­benzene­sulfonamide monohydrate (Purandara *et al.*, 2015 ▸).

## Synthesis and crystallization   

*p*-Toluene­sulfonyl chloride (0.01 mol) was added to glycine (0.02 mol) dissolved in an aqueous solution of potassium carbonate (0.06 mol, 50 ml). The reaction mixture was stirred at 373 K for 6 h, left overnight at room temperature, then filtered and treated with dilute hydro­chloric acid. The solid *N*-(*p*-toluene­sulfon­yl)glycine (*L*1) obtained was crystallized from aqueous ethanol.

Sulfuric acid (0.5 ml) was added to *L*1 (0.02 mol) dissolved in ethanol (30 ml) and the mixture was refluxed. The reaction was monitored by TLC at regular inter­vals. After completion of the reaction, the reaction mixture was concentrated to remove the excess ethanol. The product, *N*-(*p*-toluene­sulfon­yl)glycine ethyl ester (*L*2) was poured into water, neutralized with sodium bicarbonate and recrystallized from acetone.

The pure *L*2 (0.01 mol) was then added in small portions to a stirred solution of 99% hydrazine hydrate (10 ml) in 30 ml ethanol and the mixture was refluxed for 6 h. After cooling to room temperature, the resulting precipitate was filtered, washed with cold water and dried to give *N*-(*p*-toluene­sulfon­yl)glycinyl hydrazide (*L*3).

A mixture of *L*3 (0.01 mol) and *p*-methyl­benzaldehyde (0.01 mol) in anhydrous methanol (30 ml) and two drops of glacial acetic acid was refluxed for 8 h. After cooling, the precipitate was collected by vacuum filtration, washed with cold methanol and dried. It was recrystallized to constant melting point from methanol (455–457 K). Prism-like colourless single crystals were grown from a DMF solution by slow evaporation of the solvent. The purity of the compound was checked by TLC and characterized by its IR spectrum. The characteristic absorptions observed are 3286.7, 1678.1, 1606.7, 1323.2 and 1157.3 cm^−1^ for the stretching bands of N—H, C—O, C—N, S—O asymmetric and S—O symmetric, respectively.

## Refinement   

Crystal data, data collection and structure refinement details are summarized in Table 2 ▸. The amino H atoms were located in difference Fourier maps and refined with distance restraints: N—H = 0.86 (2) Å with *U*_iso_(H) = 1.2*U*_eq_(N). The C-bound H atoms were positioned with idealized geometry and refined using a riding model: C—H = 0.93–0.97 Å with *U*_iso_(H) = 1.5*U*_eq_(C) for methyl H atoms and 1.2*U*_eq_(C) for other H atoms.

## Supplementary Material

Crystal structure: contains datablock(s) I, global. DOI: 10.1107/S2056989015009330/su5131sup1.cif

Structure factors: contains datablock(s) I. DOI: 10.1107/S2056989015009330/su5131Isup2.hkl

Supporting information file. DOI: 10.1107/S2056989015009330/su5131Isup3.cml

CCDC reference: 1401257

Additional supporting information:  crystallographic information; 3D view; checkCIF report

## Figures and Tables

**Table 1:** Hydrogen-bond geometry (, ) *Cg*1 and *Cg*3 are the centroids of the *p*-toluenesulfonamide rings C1C6 and C18C23, respectively.

*D*H*A*	*D*H	H*A*	*D* *A*	*D*H*A*
N1H1*N*O2^i^	0.84(2)	2.13(2)	2.947(2)	162(2)
N2H2*N*O6^ii^	0.85(2)	2.21(2)	3.047(3)	169(2)
N4H4*N*O2^iii^	0.83(2)	2.18(2)	2.965(3)	157(3)
N5H5*N*O3^iv^	0.86(2)	1.96(2)	2.809(3)	169(3)
C6H6O6^v^	0.93	2.55	3.305(3)	139
C7H7*A*O5^v^	0.97	2.51	3.256(3)	133
C19H19O4^v^	0.93	2.57	3.212(4)	127
C14H14*Cg*1^vi^	0.93	2.91	3.832(3)	171
C29H29*Cg*3^iv^	0.93	2.84	3.753(4)	167

Symmetry codes: (i) 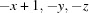; (ii) 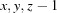; (iii) 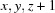; (iv) 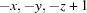; (v) 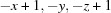; (vi) 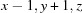.

**Table 2:** Experimental details

Crystal data	
Chemical formula	C_17_H_19_N_3_O_3_S
*M*_r_	345.41
Crystal system, space group	Triclinic, *P* 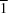
Temperature (K)	293
*a*, *b*, *c* ()	11.2595(7), 11.2697(9), 14.538(1)
, , ()	70.562(6), 87.330(7), 82.262(6)
*V* (^3^)	1723.8(2)
*Z*	4
Radiation type	Mo *K*
(mm^1^)	0.21
Crystal size (mm)	0.36 0.28 0.24

Data collection	
Diffractometer	Oxford Diffraction Xcalibur with a Sapphire CCD detector
Absorption correction	Multi-scan (*CrysAlis RED*; Oxford Diffraction, 2009 ▸)
*T*_min_, *T*_max_	0.929, 0.952
No. of measured, independent and observed [*I* > 2(*I*)] reflections	11371, 6281, 4859
*R*_int_	0.020
(sin /)_max_ (^1^)	0.602

Refinement	
*R*[*F* ^2^ > 2(*F* ^2^)], *wR*(*F* ^2^), *S*	0.047, 0.115, 1.07
No. of reflections	6281
No. of parameters	449
No. of restraints	4
H-atom treatment	H atoms treated by a mixture of independent and constrained refinement
_max_, _min_ (e ^3^)	0.21, 0.37

Computer programs: *CrysAlis CCD* and *CrysAlis RED* (Oxford Diffraction, 2009 ▸), *SHELXS97* and *SHELXL97* (Sheldrick, 2008 ▸), *PLATON* (Spek, 2009 ▸) and *Mercury* (Macrae *et al.*, 2008 ▸).

## supplementary crystallographic information

### Crystal data

**Table:**

C_17_H_19_N_3_O_3_S	*Z* = 4
*M**_r_* = 345.41	*F*(000) = 728
Triclinic, *P*1	*D*_x_ = 1.331 Mg m^−^^3^
Hall symbol: -P 1	Mo *K*α radiation, λ = 0.71073 Å
*a* = 11.2595 (7) Å	Cell parameters from 4653 reflections
*b* = 11.2697 (9) Å	θ = 2.5–27.9°
*c* = 14.538 (1) Å	µ = 0.21 mm^−^^1^
α = 70.562 (6)°	*T* = 293 K
β = 87.330 (7)°	Prism, colourless
γ = 82.262 (6)°	0.36 × 0.28 × 0.24 mm
*V* = 1723.8 (2) Å^3^	

### Data collection

**Table:**

Oxford Diffraction Xcalibur single crystal X-ray diffractometer with a Sapphire CCD detector	6281 independent reflections
Radiation source: fine-focus sealed tube	4859 reflections with *I* > 2σ(*I*)
Graphite monochromator	*R*_int_ = 0.020
Rotation method data acquisition using ω scans	θ_max_ = 25.4°, θ_min_ = 2.5°
Absorption correction: multi-scan (*CrysAlis RED*; Oxford Diffraction, 2009)	*h* = −13→11
*T*_min_ = 0.929, *T*_max_ = 0.952	*k* = −12→13
11371 measured reflections	*l* = −17→17

### Refinement

**Table:**

Refinement on *F*^2^	Primary atom site location: structure-invariant direct methods
Least-squares matrix: full	Secondary atom site location: difference Fourier map
*R*[*F*^2^ > 2σ(*F*^2^)] = 0.047	Hydrogen site location: inferred from neighbouring sites
*wR*(*F*^2^) = 0.115	H atoms treated by a mixture of independent and constrained refinement
*S* = 1.07	*w* = 1/[σ^2^(*F*_o_^2^) + (0.0366*P*)^2^ + 1.1753*P*] where *P* = (*F*_o_^2^ + 2*F*_c_^2^)/3
6281 reflections	(Δ/σ)_max_ = 0.016
449 parameters	Δρ_max_ = 0.21 e Å^−^^3^
4 restraints	Δρ_min_ = −0.37 e Å^−^^3^

### Special details

**Table:**

Geometry. All e.s.d.'s (except the e.s.d. in the dihedral angle between two l.s. planes) are estimated using the full covariance matrix. The cell e.s.d.'s are taken into account individually in the estimation of e.s.d.'s in distances, angles and torsion angles; correlations between e.s.d.'s in cell parameters are only used when they are defined by crystal symmetry. An approximate (isotropic) treatment of cell e.s.d.'s is used for estimating e.s.d.'s involving l.s. planes.
Refinement. Refinement of *F*^2^ against ALL reflections. The weighted *R*-factor *wR* and goodness of fit *S* are based on *F*^2^, conventional *R*-factors *R* are based on *F*, with *F* set to zero for negative *F*^2^. The threshold expression of *F*^2^ > σ(*F*^2^) is used only for calculating *R*-factors(gt) *etc*. and is not relevant to the choice of reflections for refinement. *R*-factors based on *F*^2^ are statistically about twice as large as those based on *F*, and *R*- factors based on ALL data will be even larger.

### Fractional atomic coordinates and isotropic or equivalent isotropic displacement parameters (Å^2^)

**Table:**

	*x*	*y*	*z*	*U*_iso_*/*U*_eq_	
S1	0.43117 (5)	−0.17537 (5)	−0.01465 (4)	0.03681 (15)	
O1	0.36089 (15)	−0.22646 (16)	−0.06749 (12)	0.0489 (4)	
O2	0.51332 (14)	−0.08895 (15)	−0.06644 (12)	0.0453 (4)	
O3	0.07623 (16)	−0.07163 (18)	0.17483 (13)	0.0594 (5)	
N1	0.34260 (16)	−0.09801 (18)	0.04163 (15)	0.0392 (4)	
H1N	0.381 (2)	−0.053 (2)	0.0626 (17)	0.047*	
N2	0.13018 (17)	0.04983 (19)	0.02447 (15)	0.0434 (5)	
H2N	0.171 (2)	0.053 (2)	−0.0265 (15)	0.052*	
N3	0.04565 (17)	0.15249 (19)	0.02068 (15)	0.0445 (5)	
C1	0.51424 (19)	−0.3020 (2)	0.07298 (16)	0.0355 (5)	
C2	0.4918 (2)	−0.4252 (2)	0.09054 (18)	0.0464 (6)	
H2	0.4308	−0.4418	0.0573	0.056*	
C3	0.5606 (3)	−0.5233 (2)	0.15777 (19)	0.0544 (7)	
H3	0.5467	−0.6063	0.1686	0.065*	
C4	0.6499 (2)	−0.5007 (3)	0.20942 (19)	0.0533 (7)	
C5	0.6692 (2)	−0.3763 (3)	0.19142 (19)	0.0521 (6)	
H5	0.7286	−0.3595	0.2260	0.062*	
C6	0.6032 (2)	−0.2771 (2)	0.12386 (17)	0.0439 (6)	
H6	0.6180	−0.1942	0.1124	0.053*	
C7	0.2439 (2)	−0.1537 (2)	0.10092 (18)	0.0423 (5)	
H7A	0.2721	−0.2001	0.1668	0.051*	
H7B	0.2158	−0.2134	0.0745	0.051*	
C8	0.1414 (2)	−0.0541 (2)	0.10389 (17)	0.0408 (5)	
C9	0.0466 (2)	0.2452 (2)	−0.05851 (19)	0.0447 (6)	
H9	0.0995	0.2364	−0.1076	0.054*	
C10	−0.0314 (2)	0.3642 (2)	−0.07566 (18)	0.0412 (5)	
C11	−0.0239 (2)	0.4593 (3)	−0.16435 (19)	0.0540 (7)	
H11	0.0298	0.4452	−0.2113	0.065*	
C12	−0.0944 (3)	0.5742 (3)	−0.1843 (2)	0.0599 (7)	
H12	−0.0873	0.6366	−0.2445	0.072*	
C13	−0.1752 (2)	0.5988 (2)	−0.1170 (2)	0.0505 (6)	
C14	−0.1822 (2)	0.5038 (3)	−0.0284 (2)	0.0530 (7)	
H14	−0.2360	0.5183	0.0183	0.064*	
C15	−0.1120 (2)	0.3886 (2)	−0.00723 (19)	0.0473 (6)	
H15	−0.1186	0.3267	0.0533	0.057*	
C16	0.7256 (3)	−0.6088 (3)	0.2820 (3)	0.0899 (11)	
H16A	0.7480	−0.6756	0.2549	0.135*	
H16B	0.6805	−0.6403	0.3408	0.135*	
H16C	0.7964	−0.5795	0.2963	0.135*	
C17	−0.2530 (3)	0.7248 (3)	−0.1391 (3)	0.0770 (9)	
H17A	−0.3310	0.7174	−0.1591	0.115*	
H17B	−0.2171	0.7877	−0.1906	0.115*	
H17C	−0.2604	0.7494	−0.0817	0.115*	
S2	0.55311 (6)	0.09396 (8)	0.65448 (5)	0.0589 (2)	
O4	0.59893 (19)	−0.0024 (2)	0.61467 (17)	0.0774 (6)	
O5	0.63233 (18)	0.1381 (2)	0.70615 (17)	0.0843 (7)	
O6	0.24349 (16)	0.07650 (17)	0.82524 (13)	0.0546 (5)	
N4	0.4440 (2)	0.0538 (3)	0.72822 (16)	0.0628 (7)	
H4N	0.460 (3)	0.035 (3)	0.7870 (14)	0.075*	
N5	0.13783 (18)	−0.0168 (2)	0.74840 (15)	0.0457 (5)	
H5N	0.0720 (18)	0.000 (2)	0.7769 (18)	0.055*	
N6	0.13391 (17)	−0.07035 (18)	0.67618 (14)	0.0415 (5)	
C18	0.4840 (2)	0.2221 (3)	0.55871 (19)	0.0528 (7)	
C19	0.4764 (3)	0.2135 (3)	0.4672 (2)	0.0648 (8)	
H19	0.5095	0.1401	0.4548	0.078*	
C20	0.4195 (3)	0.3142 (3)	0.3940 (2)	0.0727 (9)	
H20	0.4146	0.3073	0.3324	0.087*	
C21	0.3698 (3)	0.4244 (3)	0.4090 (2)	0.0680 (8)	
C22	0.3772 (3)	0.4304 (3)	0.5017 (3)	0.0737 (9)	
H22	0.3432	0.5034	0.5142	0.088*	
C23	0.4334 (3)	0.3316 (3)	0.5761 (2)	0.0675 (8)	
H23	0.4374	0.3381	0.6379	0.081*	
C24	0.3458 (2)	−0.0013 (3)	0.70435 (18)	0.0488 (6)	
H24A	0.3676	−0.0919	0.7187	0.059*	
H24B	0.3272	0.0366	0.6354	0.059*	
C25	0.2387 (2)	0.0232 (2)	0.76443 (17)	0.0423 (5)	
C26	0.0308 (2)	−0.0913 (2)	0.65790 (17)	0.0422 (5)	
H26	−0.0352	−0.0703	0.6928	0.051*	
C27	0.0140 (2)	−0.1478 (2)	0.58330 (16)	0.0404 (5)	
C28	−0.0982 (2)	−0.1755 (2)	0.56938 (18)	0.0497 (6)	
H28	−0.1627	−0.1557	0.6063	0.060*	
C29	−0.1157 (3)	−0.2325 (3)	0.50103 (19)	0.0559 (7)	
H29	−0.1916	−0.2520	0.4936	0.067*	
C30	−0.0230 (3)	−0.2610 (2)	0.44395 (19)	0.0552 (7)	
C31	0.0883 (2)	−0.2282 (3)	0.4552 (2)	0.0566 (7)	
H31	0.1516	−0.2437	0.4156	0.068*	
C32	0.1069 (2)	−0.1732 (2)	0.52392 (18)	0.0489 (6)	
H32	0.1826	−0.1528	0.5306	0.059*	
C33	0.3084 (4)	0.5341 (4)	0.3279 (3)	0.0983 (12)	
H33A	0.2243	0.5456	0.3419	0.147*	
H33B	0.3195	0.5168	0.2675	0.147*	
H33C	0.3422	0.6099	0.3226	0.147*	
C34	−0.0421 (3)	−0.3240 (3)	0.3695 (2)	0.0814 (10)	
H34A	−0.1259	−0.3127	0.3547	0.122*	
H34B	−0.0140	−0.4130	0.3955	0.122*	
H34C	0.0016	−0.2863	0.3110	0.122*	

### Atomic displacement parameters (Å^2^)

**Table:**

	*U*^11^	*U*^22^	*U*^33^	*U*^12^	*U*^13^	*U*^23^
S1	0.0365 (3)	0.0364 (3)	0.0384 (3)	−0.0033 (2)	−0.0001 (2)	−0.0141 (2)
O1	0.0531 (10)	0.0495 (10)	0.0487 (10)	−0.0065 (8)	−0.0111 (8)	−0.0209 (8)
O2	0.0442 (9)	0.0439 (9)	0.0455 (9)	−0.0082 (7)	0.0067 (7)	−0.0116 (8)
O3	0.0507 (11)	0.0712 (13)	0.0507 (11)	0.0006 (9)	0.0139 (9)	−0.0177 (9)
N1	0.0318 (10)	0.0384 (11)	0.0522 (12)	−0.0059 (8)	0.0004 (9)	−0.0210 (9)
N2	0.0390 (11)	0.0467 (12)	0.0453 (12)	0.0019 (9)	0.0053 (9)	−0.0196 (10)
N3	0.0360 (11)	0.0466 (12)	0.0549 (13)	0.0007 (9)	−0.0010 (9)	−0.0242 (11)
C1	0.0361 (12)	0.0356 (12)	0.0360 (12)	−0.0014 (9)	0.0029 (9)	−0.0151 (10)
C2	0.0530 (15)	0.0435 (14)	0.0464 (14)	−0.0078 (12)	−0.0003 (11)	−0.0190 (12)
C3	0.0699 (18)	0.0355 (14)	0.0550 (16)	−0.0044 (13)	0.0057 (14)	−0.0126 (12)
C4	0.0507 (15)	0.0528 (16)	0.0455 (15)	0.0076 (13)	0.0030 (12)	−0.0074 (12)
C5	0.0432 (14)	0.0616 (17)	0.0505 (15)	−0.0024 (12)	−0.0064 (12)	−0.0182 (13)
C6	0.0420 (13)	0.0426 (13)	0.0500 (14)	−0.0041 (11)	−0.0034 (11)	−0.0191 (12)
C7	0.0374 (12)	0.0408 (13)	0.0485 (14)	−0.0068 (10)	0.0045 (10)	−0.0143 (11)
C8	0.0343 (12)	0.0492 (14)	0.0438 (14)	−0.0076 (11)	0.0012 (10)	−0.0211 (12)
C9	0.0383 (13)	0.0510 (15)	0.0483 (15)	−0.0034 (11)	0.0004 (11)	−0.0222 (13)
C10	0.0340 (12)	0.0454 (14)	0.0498 (14)	−0.0044 (10)	−0.0031 (10)	−0.0228 (12)
C11	0.0540 (16)	0.0580 (17)	0.0487 (15)	0.0035 (13)	0.0081 (12)	−0.0210 (13)
C12	0.0679 (18)	0.0546 (17)	0.0483 (16)	0.0043 (14)	0.0018 (13)	−0.0101 (13)
C13	0.0474 (15)	0.0528 (15)	0.0562 (16)	0.0019 (12)	−0.0058 (12)	−0.0270 (13)
C14	0.0457 (15)	0.0632 (17)	0.0570 (16)	−0.0037 (13)	0.0086 (12)	−0.0315 (14)
C15	0.0458 (14)	0.0494 (15)	0.0480 (15)	−0.0073 (12)	0.0044 (11)	−0.0180 (12)
C16	0.079 (2)	0.072 (2)	0.089 (3)	0.0150 (18)	−0.0154 (19)	0.0056 (19)
C17	0.080 (2)	0.065 (2)	0.084 (2)	0.0192 (17)	−0.0088 (18)	−0.0318 (18)
S2	0.0454 (4)	0.0787 (5)	0.0626 (4)	−0.0215 (4)	0.0047 (3)	−0.0320 (4)
O4	0.0655 (13)	0.0849 (15)	0.0886 (16)	0.0003 (11)	0.0035 (11)	−0.0419 (13)
O5	0.0582 (13)	0.1185 (19)	0.0939 (16)	−0.0379 (13)	−0.0071 (11)	−0.0473 (15)
O6	0.0592 (11)	0.0601 (11)	0.0549 (11)	−0.0050 (9)	−0.0064 (9)	−0.0329 (9)
N4	0.0575 (14)	0.0940 (18)	0.0423 (12)	−0.0346 (13)	−0.0014 (11)	−0.0199 (13)
N5	0.0391 (11)	0.0578 (13)	0.0479 (12)	−0.0048 (10)	0.0018 (9)	−0.0284 (10)
N6	0.0429 (11)	0.0435 (11)	0.0403 (11)	−0.0059 (9)	−0.0022 (9)	−0.0163 (9)
C18	0.0505 (15)	0.0647 (17)	0.0537 (16)	−0.0263 (13)	0.0157 (12)	−0.0280 (14)
C19	0.081 (2)	0.0655 (19)	0.0554 (18)	−0.0194 (16)	0.0181 (15)	−0.0285 (16)
C20	0.092 (2)	0.079 (2)	0.0512 (18)	−0.0248 (19)	0.0127 (16)	−0.0239 (17)
C21	0.0654 (19)	0.070 (2)	0.070 (2)	−0.0253 (16)	0.0111 (16)	−0.0209 (17)
C22	0.075 (2)	0.067 (2)	0.089 (2)	−0.0143 (17)	0.0110 (18)	−0.0383 (19)
C23	0.074 (2)	0.080 (2)	0.0650 (19)	−0.0208 (17)	0.0095 (16)	−0.0428 (18)
C24	0.0468 (14)	0.0583 (16)	0.0448 (14)	−0.0159 (12)	−0.0002 (11)	−0.0182 (12)
C25	0.0464 (14)	0.0392 (13)	0.0411 (13)	−0.0029 (11)	−0.0050 (10)	−0.0134 (11)
C26	0.0412 (13)	0.0427 (13)	0.0408 (13)	−0.0038 (11)	0.0006 (10)	−0.0121 (11)
C27	0.0434 (13)	0.0367 (12)	0.0381 (12)	−0.0059 (10)	−0.0056 (10)	−0.0074 (10)
C28	0.0464 (14)	0.0575 (16)	0.0431 (14)	−0.0148 (12)	−0.0003 (11)	−0.0108 (12)
C29	0.0565 (16)	0.0613 (17)	0.0511 (16)	−0.0252 (14)	−0.0086 (13)	−0.0123 (13)
C30	0.0702 (18)	0.0483 (15)	0.0493 (15)	−0.0094 (13)	−0.0121 (14)	−0.0167 (13)
C31	0.0550 (16)	0.0619 (17)	0.0578 (17)	0.0008 (14)	−0.0023 (13)	−0.0292 (14)
C32	0.0412 (13)	0.0547 (15)	0.0542 (15)	−0.0033 (12)	−0.0063 (11)	−0.0228 (13)
C33	0.098 (3)	0.089 (3)	0.098 (3)	−0.007 (2)	−0.008 (2)	−0.018 (2)
C34	0.104 (3)	0.081 (2)	0.074 (2)	−0.017 (2)	−0.0168 (19)	−0.0415 (19)

### Geometric parameters (Å, º)

**Table:**

S1—O1	1.4218 (16)	S2—O4	1.421 (2)
S1—O2	1.4355 (16)	S2—O5	1.427 (2)
S1—N1	1.6090 (19)	S2—N4	1.602 (2)
S1—C1	1.754 (2)	S2—C18	1.758 (3)
O3—C8	1.214 (3)	O6—C25	1.229 (3)
N1—C7	1.453 (3)	N4—C24	1.447 (3)
N1—H1N	0.846 (16)	N4—H4N	0.831 (17)
N2—C8	1.339 (3)	N5—C25	1.334 (3)
N2—N3	1.383 (3)	N5—N6	1.379 (3)
N2—H2N	0.850 (16)	N5—H5N	0.861 (16)
N3—C9	1.272 (3)	N6—C26	1.272 (3)
C1—C2	1.382 (3)	C18—C19	1.373 (4)
C1—C6	1.383 (3)	C18—C23	1.383 (4)
C2—C3	1.378 (4)	C19—C20	1.376 (4)
C2—H2	0.9300	C19—H19	0.9300
C3—C4	1.380 (4)	C20—C21	1.374 (4)
C3—H3	0.9300	C20—H20	0.9300
C4—C5	1.383 (4)	C21—C22	1.378 (4)
C4—C16	1.507 (4)	C21—C33	1.508 (5)
C5—C6	1.372 (3)	C22—C23	1.372 (4)
C5—H5	0.9300	C22—H22	0.9300
C6—H6	0.9300	C23—H23	0.9300
C7—C8	1.508 (3)	C24—C25	1.506 (3)
C7—H7A	0.9700	C24—H24A	0.9700
C7—H7B	0.9700	C24—H24B	0.9700
C9—C10	1.454 (3)	C26—C27	1.459 (3)
C9—H9	0.9300	C26—H26	0.9300
C10—C11	1.383 (3)	C27—C28	1.381 (3)
C10—C15	1.385 (3)	C27—C32	1.386 (3)
C11—C12	1.373 (4)	C28—C29	1.384 (4)
C11—H11	0.9300	C28—H28	0.9300
C12—C13	1.375 (4)	C29—C30	1.373 (4)
C12—H12	0.9300	C29—H29	0.9300
C13—C14	1.381 (4)	C30—C31	1.385 (4)
C13—C17	1.507 (4)	C30—C34	1.515 (4)
C14—C15	1.373 (3)	C31—C32	1.375 (3)
C14—H14	0.9300	C31—H31	0.9300
C15—H15	0.9300	C32—H32	0.9300
C16—H16A	0.9600	C33—H33A	0.9600
C16—H16B	0.9600	C33—H33B	0.9600
C16—H16C	0.9600	C33—H33C	0.9600
C17—H17A	0.9600	C34—H34A	0.9600
C17—H17B	0.9600	C34—H34B	0.9600
C17—H17C	0.9600	C34—H34C	0.9600
			
O1—S1—O2	119.33 (10)	O4—S2—O5	119.00 (14)
O1—S1—N1	108.63 (10)	O4—S2—N4	112.07 (14)
O2—S1—N1	104.92 (10)	O5—S2—N4	104.85 (13)
O1—S1—C1	108.15 (10)	O4—S2—C18	107.17 (13)
O2—S1—C1	107.25 (10)	O5—S2—C18	109.91 (14)
N1—S1—C1	108.09 (10)	N4—S2—C18	102.65 (13)
C7—N1—S1	121.38 (15)	C24—N4—S2	122.13 (18)
C7—N1—H1N	117.5 (17)	C24—N4—H4N	117 (2)
S1—N1—H1N	110.3 (17)	S2—N4—H4N	115 (2)
C8—N2—N3	121.0 (2)	C25—N5—N6	120.1 (2)
C8—N2—H2N	121.0 (18)	C25—N5—H5N	120.8 (18)
N3—N2—H2N	117.9 (18)	N6—N5—H5N	118.5 (18)
C9—N3—N2	114.1 (2)	C26—N6—N5	115.8 (2)
C2—C1—C6	120.4 (2)	C19—C18—C23	119.4 (3)
C2—C1—S1	120.50 (18)	C19—C18—S2	120.7 (2)
C6—C1—S1	119.12 (17)	C23—C18—S2	119.9 (2)
C3—C2—C1	119.3 (2)	C18—C19—C20	119.5 (3)
C3—C2—H2	120.3	C18—C19—H19	120.2
C1—C2—H2	120.3	C20—C19—H19	120.2
C2—C3—C4	121.3 (2)	C21—C20—C19	122.2 (3)
C2—C3—H3	119.3	C21—C20—H20	118.9
C4—C3—H3	119.3	C19—C20—H20	118.9
C3—C4—C5	118.1 (2)	C20—C21—C22	117.2 (3)
C3—C4—C16	120.8 (3)	C20—C21—C33	121.6 (3)
C5—C4—C16	121.0 (3)	C22—C21—C33	121.2 (3)
C6—C5—C4	121.8 (2)	C23—C22—C21	121.8 (3)
C6—C5—H5	119.1	C23—C22—H22	119.1
C4—C5—H5	119.1	C21—C22—H22	119.1
C5—C6—C1	119.1 (2)	C22—C23—C18	119.7 (3)
C5—C6—H6	120.4	C22—C23—H23	120.1
C1—C6—H6	120.4	C18—C23—H23	120.1
N1—C7—C8	111.69 (19)	N4—C24—C25	108.2 (2)
N1—C7—H7A	109.3	N4—C24—H24A	110.1
C8—C7—H7A	109.3	C25—C24—H24A	110.1
N1—C7—H7B	109.3	N4—C24—H24B	110.1
C8—C7—H7B	109.3	C25—C24—H24B	110.1
H7A—C7—H7B	107.9	H24A—C24—H24B	108.4
O3—C8—N2	124.9 (2)	O6—C25—N5	121.6 (2)
O3—C8—C7	120.0 (2)	O6—C25—C24	122.2 (2)
N2—C8—C7	115.1 (2)	N5—C25—C24	116.2 (2)
N3—C9—C10	122.9 (2)	N6—C26—C27	121.4 (2)
N3—C9—H9	118.6	N6—C26—H26	119.3
C10—C9—H9	118.6	C27—C26—H26	119.3
C11—C10—C15	117.9 (2)	C28—C27—C32	118.1 (2)
C11—C10—C9	118.6 (2)	C28—C27—C26	119.6 (2)
C15—C10—C9	123.5 (2)	C32—C27—C26	122.3 (2)
C12—C11—C10	121.2 (2)	C27—C28—C29	120.7 (2)
C12—C11—H11	119.4	C27—C28—H28	119.7
C10—C11—H11	119.4	C29—C28—H28	119.7
C11—C12—C13	121.3 (3)	C30—C29—C28	121.3 (2)
C11—C12—H12	119.4	C30—C29—H29	119.4
C13—C12—H12	119.4	C28—C29—H29	119.4
C12—C13—C14	117.5 (2)	C29—C30—C31	117.9 (2)
C12—C13—C17	121.2 (3)	C29—C30—C34	121.2 (3)
C14—C13—C17	121.3 (3)	C31—C30—C34	120.8 (3)
C15—C14—C13	122.0 (2)	C32—C31—C30	121.2 (3)
C15—C14—H14	119.0	C32—C31—H31	119.4
C13—C14—H14	119.0	C30—C31—H31	119.4
C14—C15—C10	120.2 (2)	C31—C32—C27	120.7 (2)
C14—C15—H15	119.9	C31—C32—H32	119.6
C10—C15—H15	119.9	C27—C32—H32	119.6
C4—C16—H16A	109.5	C21—C33—H33A	109.5
C4—C16—H16B	109.5	C21—C33—H33B	109.5
H16A—C16—H16B	109.5	H33A—C33—H33B	109.5
C4—C16—H16C	109.5	C21—C33—H33C	109.5
H16A—C16—H16C	109.5	H33A—C33—H33C	109.5
H16B—C16—H16C	109.5	H33B—C33—H33C	109.5
C13—C17—H17A	109.5	C30—C34—H34A	109.5
C13—C17—H17B	109.5	C30—C34—H34B	109.5
H17A—C17—H17B	109.5	H34A—C34—H34B	109.5
C13—C17—H17C	109.5	C30—C34—H34C	109.5
H17A—C17—H17C	109.5	H34A—C34—H34C	109.5
H17B—C17—H17C	109.5	H34B—C34—H34C	109.5
			
O1—S1—N1—C7	49.8 (2)	O4—S2—N4—C24	−47.0 (3)
O2—S1—N1—C7	178.47 (18)	O5—S2—N4—C24	−177.4 (2)
C1—S1—N1—C7	−67.3 (2)	C18—S2—N4—C24	67.7 (3)
C8—N2—N3—C9	178.9 (2)	C25—N5—N6—C26	−171.7 (2)
O1—S1—C1—C2	−9.3 (2)	O4—S2—C18—C19	3.5 (3)
O2—S1—C1—C2	−139.20 (19)	O5—S2—C18—C19	134.1 (2)
N1—S1—C1—C2	108.2 (2)	N4—S2—C18—C19	−114.7 (2)
O1—S1—C1—C6	170.28 (18)	O4—S2—C18—C23	−178.6 (2)
O2—S1—C1—C6	40.4 (2)	O5—S2—C18—C23	−47.9 (3)
N1—S1—C1—C6	−72.3 (2)	N4—S2—C18—C23	63.2 (2)
C6—C1—C2—C3	−1.4 (4)	C23—C18—C19—C20	0.6 (4)
S1—C1—C2—C3	178.15 (18)	S2—C18—C19—C20	178.6 (2)
C1—C2—C3—C4	1.4 (4)	C18—C19—C20—C21	0.2 (5)
C2—C3—C4—C5	−0.4 (4)	C19—C20—C21—C22	−0.9 (5)
C2—C3—C4—C16	−179.3 (3)	C19—C20—C21—C33	179.6 (3)
C3—C4—C5—C6	−0.6 (4)	C20—C21—C22—C23	1.0 (5)
C16—C4—C5—C6	178.3 (3)	C33—C21—C22—C23	−179.6 (3)
C4—C5—C6—C1	0.5 (4)	C21—C22—C23—C18	−0.2 (5)
C2—C1—C6—C5	0.5 (3)	C19—C18—C23—C22	−0.6 (4)
S1—C1—C6—C5	−179.10 (18)	S2—C18—C23—C22	−178.6 (2)
S1—N1—C7—C8	−151.08 (17)	S2—N4—C24—C25	−156.3 (2)
N3—N2—C8—O3	5.1 (4)	N6—N5—C25—O6	176.7 (2)
N3—N2—C8—C7	−175.06 (19)	N6—N5—C25—C24	−3.6 (3)
N1—C7—C8—O3	−150.8 (2)	N4—C24—C25—O6	−3.1 (3)
N1—C7—C8—N2	29.3 (3)	N4—C24—C25—N5	177.2 (2)
N2—N3—C9—C10	−177.2 (2)	N5—N6—C26—C27	−179.9 (2)
N3—C9—C10—C11	−179.8 (2)	N6—C26—C27—C28	176.4 (2)
N3—C9—C10—C15	1.2 (4)	N6—C26—C27—C32	−4.7 (4)
C15—C10—C11—C12	−0.3 (4)	C32—C27—C28—C29	3.0 (4)
C9—C10—C11—C12	−179.3 (2)	C26—C27—C28—C29	−178.1 (2)
C10—C11—C12—C13	−0.2 (4)	C27—C28—C29—C30	−1.2 (4)
C11—C12—C13—C14	0.4 (4)	C28—C29—C30—C31	−1.5 (4)
C11—C12—C13—C17	−179.7 (3)	C28—C29—C30—C34	179.7 (3)
C12—C13—C14—C15	−0.2 (4)	C29—C30—C31—C32	2.4 (4)
C17—C13—C14—C15	180.0 (3)	C34—C30—C31—C32	−178.7 (3)
C13—C14—C15—C10	−0.3 (4)	C30—C31—C32—C27	−0.6 (4)
C11—C10—C15—C14	0.5 (4)	C28—C27—C32—C31	−2.0 (4)
C9—C10—C15—C14	179.5 (2)	C26—C27—C32—C31	179.0 (2)

### Hydrogen-bond geometry (Å, º)

Cg1 and Cg3 are the centroids of the *p*-toluenesulfonamide rings C1–C6 and C18–C23, respectively.

**Table:**

*D*—H···*A*	*D*—H	H···*A*	*D*···*A*	*D*—H···*A*
N1—H1*N*···O2^i^	0.84 (2)	2.13 (2)	2.947 (2)	162 (2)
N2—H2*N*···O6^ii^	0.85 (2)	2.21 (2)	3.047 (3)	169 (2)
N4—H4*N*···O2^iii^	0.83 (2)	2.18 (2)	2.965 (3)	157 (3)
N5—H5*N*···O3^iv^	0.86 (2)	1.96 (2)	2.809 (3)	169 (3)
C6—H6···O6^v^	0.93	2.55	3.305 (3)	139
C7—H7*A*···O5^v^	0.97	2.51	3.256 (3)	133
C19—H19···O4^v^	0.93	2.57	3.212 (4)	127
C14—H14···*Cg*1^vi^	0.93	2.91	3.832 (3)	171
C29—H29···*Cg*3^iv^	0.93	2.84	3.753 (4)	167

Symmetry codes: (i) −*x*+1, −*y*, −*z*; (ii) *x*, *y*, *z*−1; (iii) *x*, *y*, *z*+1; (iv) −*x*, −*y*, −*z*+1; (v) −*x*+1, −*y*, −*z*+1; (vi) *x*−1, *y*+1, *z*.
